# Modes of transmission and genetic diversity of foamy viruses in a *Macaca tonkeana *colony

**DOI:** 10.1186/1742-4690-3-23

**Published:** 2006-04-11

**Authors:** Sara Calattini, Fanélie Wanert, Bernard Thierry, Christine Schmitt, Sylviane Bassot, Ali Saib, Nicolas Herrenschmidt, Antoine Gessain

**Affiliations:** 1 Unité d'Epidémiologie et Physiopathologie des Virus Oncogènes, Département de Virologie, Institut Pasteur, Paris, France; 2 Centre de Primatologie, et CNRS UPR 9010, Université Louis Pasteur, Strasbourg, France; 3 Platte-forme de Microscopie Electronique, Insitut Pasteur, Paris, France; 4 CNRS UMR7151, Hôpital Saint Louis, Paris, France

## Abstract

**Background:**

Foamy viruses are exogenous complex retroviruses that are highly endemic in several animal species, including monkeys and apes, where they cause persistent infection. Simian foamy viral (SFV) infection has been reported in few persons occupationally exposed to non-human primates (NHP) in zoos, primate centers and laboratories, and recently in few hunters from central Africa. Most of the epidemiological works performed among NHP populations concern cross-sectional studies without long-term follow-up. Therefore, the exact timing and the modes of transmission of SFVs remain not well known, although sexual and oral transmissions have been suspected. We have conducted a longitudinal study in a free-breeding colony of *Macaca tonkeana *in order (1) to determine the prevalence of the infection by foamy viruses, (2) to characterize molecularly the viruses infecting such animals, (3) to study their genetic variability overtime by long-term follow-up of several DNA samples in a series of specific animals, and (4) to get new insights concerning the timing and the modes of SFVs primary infection in these monkeys by combining serology and molecular means, as well as studies of familial structures and long-term behavioral observations.

**Results/conclusion:**

We first demonstrated that this colony was highly endemic for SFVs, with a clear increase of seroprevalence with age. Only 4.7% of immatures, and 43,7% of sub-adults were found seropositive, while 89.5% of adults exhibited antibodies directed against SFV. We further showed that 6 different strains of foamy viruses (exhibiting a very low intra-strain and overtime genetic variability in the *integrase *gene) are circulating within this group. This suggests a possible infection by different strains within an animal. Lastly, we provide strong evidence that foamy viruses are mostly acquired through severe bites, mainly in sub-adults or young adults. Most cases of seroconversion occur after 7 years of age; from this age individuals competed for access to sexual partners, thus increasing the likelihood of being wounded. Furthermore, all the serological and molecular data, obtained in this free-breeding colony, argue against a significant transmission of SFVs from mother or father to infants as well as between siblings.

## Background

Foamy viruses (FVs) are members of the *Spumavirus *genus of the *Retroviridae *family [1]. These exogenous complex retroviruses are highly prevalent in several animal species, including primates, felines, bovines and equines where they cause persistent infections [2-7]. Simian foamy viral (SFV) infection has also been reported in 1 to 4 % of persons occupationally exposed to non-human primates in zoos, primate centers and laboratories, mainly in Northern America but also in Europe [8-12]. Very recently, naturally acquired SFV infections have been described in few hunters living in Cameroon, central Africa [13] (and Calattini et al., in preparation) and in one person with frequent contacts with *Macaca fascicularis *in a temple in Bali, Indonesia [14].

Foamy viruses are considered as non-pathogenic in naturally or experimentally infected animals [15,16]. Furthermore, they do not seem to cause any disease in the very few humans who were accidentally infected, and who have then beneficiated of a long-term medical and biological follow-up [9,11,12,17]. This lack of pathogenicity contrasts strongly with the cytopathic effect that is seen *in vitro *in infected cell cultures, with the appearance of "foamy-like" syncitia [15,18,19].

In contrast to the HIV/SIV lentiviruses, foamy viruses exhibit a very low genetic drift *in vivo *[2,20-22]. Phylogenetic analyses have also demonstrated a species-specific distribution of foamy viruses. This indicates a long-term co-evolution of such retroviruses with their natural hosts [23]. Recently, Switzer et al. demonstrated that FVs might have co-speciated with Old World primates for at least 30 million years [24]. Such features could explain their possible lack of pathogenicity that is observed *in vivo *and the long-life persistence of the infection [4,20,21]. Worth noting is that the great majority of the viral strains yet characterized concerns African monkeys and Apes. Indeed, relatively few data are known on the variability of FVs in Asian monkeys, despite an important biodiversity of such animals, especially within the macaques species [8,24,25].

While the molecular features of foamy viruses have been extensively studied *in vitro *[15,18,19,26], only few data are available on the characteristics of FVs *in vivo*, including epidemiological determinants [3,4,16,20-22]. As an example, the timing and modes of primary infection are not well known.

The few published epidemiological studies indicate that among captive non human primate populations, antibodies seroprevalence to SFVs can reach up to 75–100% in adults [4,16,20]. Furthermore, there is only one recent study reporting the SFV seroprevalence in a free-ranging group of non-human primates (NHPs) [14]. This study concerns a group of 38 macaques living in Bali, Indonesia. However, most studies are cross-sectional works in captive animals and no long-term follow-up searching specifically for time and mode of seroconversion had been performed. Regarding the modes of infection, some studies have shown that SFVs are present at a high concentration in the saliva of infected animals [26-28] Throat mucosa has been shown to be an important site for viral replication in African green monkeys [27], and a very recent study demonstrated high levels of viral RNA in oral tissues of macaques [28]. All together, this suggests that bites, scratches and mucosal splashes can be mechanisms of transmission, at least in some animals. Other studies in captive colonies of baboons have suggested that sexual and/or mother to offspring transmission through saliva contacts can occurred [2,20].

We have conducted a study in a free-breeding colony of *Macaca tonkeana *housed in the Strasbourg Primatology Center in France. This colony was followed for more than 24 years for behavioral investigations including the study of social relationships and reproductive behaviors [29-34]. The goals of our current study were: 1) to determine the prevalence of SFV infection in this colony, 2) to characterize the viruses that infect these animals and to study their genetic variability overtime through a long-term follow-up, 3) to try to get new insights concerning the timing and modes of foamy viruses primary infection in these monkeys by combining serology and molecular means as well as studies of familial structures and long-term behavioral observations.

## Results

### Seroprevalence of foamy virus infection among the macaques colony

Fifty-six different animals (27 females and 29 males) were studied and a total of 141 samples were obtained during the longitudinal follow-up of these monkeys, which began with 4 animals in 1991 and ended in 2004. Based on their age at the moment of sampling, these animals have been classified as immatures (0–4 years old), sub-adults (5–8 years), or adults (>8 years old). All plasma/sera were tested with a western blot assay. The seroprevalence of the SFV among the *M. tonkeana *colony of the Primatology Center of Strasbourg is presented in Table 1.

**Table 1:** Epidemiological data of the 56 different studied *M. Tonkeana*. Serological and molecular results of foamy viruses in their peripheral blood.

CODE	SEX	Age (years) at the last sampling	W.B. FV*	INTEGRASE PCR	LTR PCR	I.F. HTLV	Viral load**
T2	F	36	+	+	-	+	1–10
RM	F	32	+	-	+	+	1–10
T1	M	28	+	+	-	+	1–10
T7	F	26	+	+	-	+	1–10
T4	F	25	+	+	+	+	100
T5	F	22	+	+	-	+	1–10
T6	F	22	+	-	+	+	1–10
T10	M	18	+	NA.	NA.	+	
TD3	F	15	+	+	-	+	1–10
TD1	F	13	+	+	+	+	1–10
TF2	F	13	+	+	-	+	100
TE3	F	12	-	-	-	+	
TG1	M	12	+	-	+	+	1–10
TG2	F	12	+	+	-	+	1–10
TG3	M	10	+	+	-	+	1–10
TI3	M	10	+	-	-	-	
TI4	M	10	+	+	+	+	100
T3	F	9	+	NA.	NA.	-	
TJ3	F	9	-	-	-	+	
T9	M	8	+	NA.	NA.	-	
TI1	M	8	-	-	-	+	
TI2	M	8	-	-	-	+	
TK3	M	8	+	+	-	-	1–10
Z10	M	8	+	+	-	-	1–10
TA1	M	7	+	NA.	NA.	-	
TL1	M	7	+	-	-	+	
TL3	F	7	-	-	-	+	
TM3	M	7	+	+	+	+	1–10
TK4	F	6	-	-	-	+	
TL2	F	5	-	NA.	NA.	+	
TN1	F	5	-	-	-	+	
TN3	F	5	-	-	-	-	
TN5	M	5	-	-	-	+	
TN7	M	5	+	-	-	+	
TN8	M	5	-	-	-	+	
TD2	M	4	-	NA.	NA.	+	
TM1	M	4	-	-	-	+	
TM2	M	4	-	-	-	+	
TP1	F	4	-	-	-	+	
TP2	M	4	-	-	-	+	
TE2	F	3	-	NA.	NA.	-	
TE4	F	3	-	-	-	+	
TN6	M	3	-	-	-	+	
TQ3	F	3	+	+	-	+	1–10
TQ6	F	3	-	-	-	+	
TQ9	M	3	-	-	-	+	
TR2	M	2	-	-	-	+	
TJ2	M	1	-	-	-	-	
TQ1	F	1	-	-	-	+	
TQ4	F	1	-	-	-	-	
TS1	F	1	-	-	-	+	
TS2	M	1	-	-	-	+	
TS3	F	1	-	-	-	+	
TS4	M	1	-	-	-	+	
TR1	M	<1	-	-	-	-	
TT1	M	<1	-	-	-	+	
TOT = 56							

W.B. = Western blot; I.F. = Immunofluorescence assay. NA. = Not Available

* Western blot performed with antigens derived from the BHK-21 cell line infected with the MtoT6 strain.

** Viral load express in number of copies of SFV genomes in 500 ng of total DNA.

**Figure 1:**
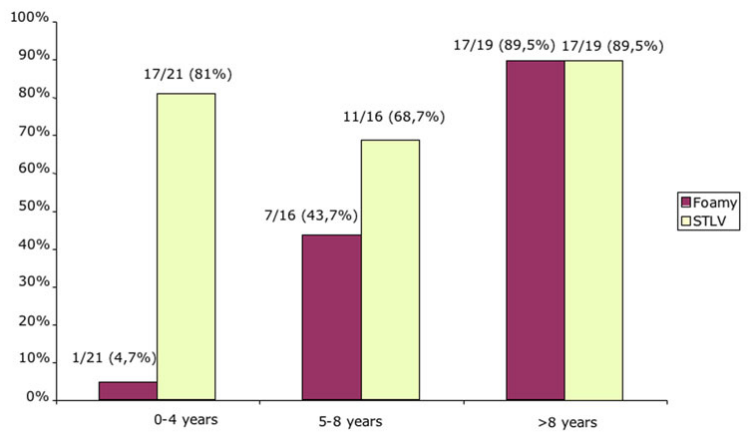
**Comparative seroprevalence rates for foamy virus and HTLV-1/STLV-1 in the 56 animals of the colony**. According to age at the last sampling, animals were classified in three groups corresponding to immatures (0–4 years old), subadults (5–8 years old) and adults (more than 8 years old).

We first performed a cross-sectional study analyzing only the last sample obtained for each animal. Twenty-five out of the 56 samples (44,6%) revealed a clear western blot sero-reactivity when screened with a BHK-21 cell line infected by a virus originating from a macaque (MtoT6) of this colony. As seen in figure 1, the rate of FVs sero-positivity increased strongly with age. Indeed, only one out of 21 immatures (4.7%), and seven out of 16 sub-adults (43,7%) were found to be SFV seropositive, while 17 out 19 adults (89.5%) exhibited antibodies directed against SFV. We then compared these data to the STLV-1/HTLV-1 serological results, obtained with the same samples. The STLV-1/HTLV-1 seroprevalence rate was already very high in the immatures animals (81%) and remained stable in the sub-adults (68.7%) and adults (89.5%) (Figure 1). Such results are consistent with the known modes of transmission for STLV-1; mainly from mother to child through breast-feeding.

In order to gain new insights on the timing of SFV infection, we undertook a longitudinal study with a long-term follow-up of this colony. Forty-one animals were tested at least twice. All of the 141 samples of the colony were tested initially with a WB using as antigen the chimpanzee foamy virus strain. Furthermore, all of the negative sera with the chimpanzee strain were then tested with a WB using antigens from the macaca foamy virus strain (BHK-21 cells infected by MtoT6). With this "autologous" virus, we found only one more positive sera (very faint seroreactivity -TN7) that was negative with the previously WB. As seen in Table 2, fourteen animals (9 adults, 5 sub-adults, and 1 immature) were found to be SFV seropositive at their first sampling. Furthermore, 17 out of the 41 animals remained SFV seronegative during the study (most of them being immatures or sub-adults), while 10 monkeys seroconverted for SFV during the follow-up.

**Table 2:** Long term serological follow-up for foamy viruses and for HTLV-1/STLV-1.

				*Year of sampling*											
				
*CODE*	*sex*	*Status at the first sampling*	*year of birth*	1991		1992		1993		1996		2002		2004	
				FV	HTLV	FV	HTLV	FV	HTLV	FV	HTLV	FV	HTLV	FV	HTLV

T2	F	A	1968	N.A.	N.A.	+	+	+	+	+	+	+	+	N.A.	N.A.
RM	F	A	1960	+	+	+	+	N.A.	N.A.	N.A.	N.A.	N.A.	N.A.	N.A.	N.A.
T1	M	A	1976	N.A.	N.A.	+	-	+	-	+	-	N.A.	N.A.	+	+
T7	F	A	1978	+	+	+	+	+	+	+	+	+	+	+	+
T4	F	A	1979	N.A.	N.A.	+	+	+	+	+	+	+	+	+	+
T5	F	A	1982	+	+	+	+	+	+	+	+	+	+	+	+
T6	F	A	1982	N.A.	N.A.	+	+	+	+	+	+	+	+	+	+
TD3	F	I	1989	N.A.	N.A.	-	N.D.	-	+	-	+	-*	+	+*	+
TD1	F	I	1989	N.A.	N.A.	+	-	+	-	+	-	+	+	N.A.	N.A.
TF2	F	I	1991			N.A.	N.A.	-	+	-*	+	+*	+	+	+
TE3	F	I	1990	N.A.	N.A.	-	N.D.	-	+	-	+	-	+	N.A.	N.A.
TG1	M	I	1992					-	+	-*	+	+*	+	+	+
TG2	F	I	1992					-*	+	+*	+	+	+	+	+
TG3	M	I	1992					N.A.	N.A.	-*	-	+*	+	N.A.	N.A.
TI3	M	I	1994							-*	-	N.A.	N.A.	+*	-
TI4	M	I	1994							-*	+	N.A.	N.A.	+*	+
T3	F	A	1984	N.A.	N.A.	+	-	+	-	N.A.	N.A.	N.A.	N.A.	N.A.	N.A.
TJ3	F	I	1995							-	N.D.	-	+	-	+
T9	M	S-A	1985	-*	+	N.A.	N.A.	+*	+	N.D.	+	N.A.	N.A.	N.A.	N.A.
TI1	M	I	1994							-	+	-	+	N.A.	N.A.
TI2	M	I	1994							-	+	-	+	N.A.	N.A.
TK3	M	S-A	1996									-*	-	+*	-
Z10	M	S-A	1996									+	-	+	-
TA1	M	S-A	1986	N.A.	N.A.	+	-	+	-	N.A.	N.A.	N.A.	N.A.	N.A.	N.A.
TL1	M	S-A	1997									+	+	+	+
TL3	F	S-A	1997									-	+	-	+
TM3	M	I	1997									+	+	+	+
TN1	F	I	1999									-	+	-	+
TN3	F	I	1999									-	-	-	-
TN5	M	I	1999									-	+	-	+
TN7	M	I	1999									-*	+	+*	+
TN8	M	I	1999									-	+	-	+
TD2	M	I	1989	N.A.	N.A.	-	N.D.	-	+	N.D.	+	N.A.	N.A.	N.A.	N.A.
TP1	F	I	2000									-	+	-	+
TP2	M	I	2000									-	+	-	+
TE2	F	I	1990	N.A.	N.A.	-	-	-	-	N.A.	N.A.	N.A.	N.A.	N.A.	N.A.
TE4	F	I	1990	N.A.	N.A.	-	N.D.	-	+	N.D.	+	N.A.	N.A.	N.A.	N.A.
TQ3	F	I	2001									+	+	+	+
TQ6	F	I	2001									-	+	-	+
TQ9	M	I	2001									-	+	-	+
TR2	M	I	2002									-	+	-	+
TOT = 41															

One hundred forty one samples of the 41 animals, for which at least two samples were obtained during the follow-up, were studied. Status at the first sampling. A = adult, S-A = subadults, I = immature. N.A. = Not Available; N.D. = Not detected. * represent the samples for which a seroconversion for foamy virus was observed during the follow-up. The Western blot were performed with antigens derived from the BHK-21 cell line infected with a chimpanzee SFV (all the samples) and from the BHK-21 cell line infected with the MtoT6 SFV strain (the last obtained sample and all the negative ones)

### Virus isolation

Isolation of SFV was assayed on five animals (T1, T5, T6, TF2 and TG1) whose WB showed a strong seropositivity. After an initial stimulation with PHA for 2 days, the PBMCs were cultured in presence of IL-2. Then, these mononuclear cells were co-cultivated with BHK-21 cells for several days with regular passages and were examined carefully for the appearance of a cytopathic effect. Giant cell formation and syncitia were first observed for the T1 sample after 8 days of co-culture, while such CPE was only detected after 12 days for the T6 and TF2 sample cells. Concerning the T5 and TG1 cells, the appearance of syncitia and giant cells was delayed until 18 days of co-culture. The destruction of the monolayer of BHK-21 was quite rapid (2 to 4 days) after the first appearance of the CPE. Regular adding of BHK-21 cells was thus necessary to sustain the culture.

**Figure 2:**
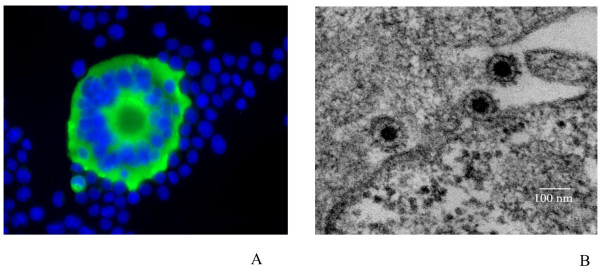
**Immunofluorescence and electron microscopy of SFV infected cells**. A. Typical multinucleated giant cells with a clear seroreactivity of MtoT1 antigens, using an immunofluorescence assay with a positive anti-foamy serum, on BHK-21 infected cells. B. Electron microscopy of ultra-thin sections from cells infected by MtoTF2. The typical foamy viral particles showed a spherical central core and several envelope spikes. The budding observed here is from the cellular membrane

In order to search for foamy viral expression, IFA was performed, using a specific anti foamy sera, on the co-cultures showing a typical CPE. Syncitia and large cells showed a strong and clear specific fluorescence (as shown in figure 2A), while negative control cells and co-culture without any CPE were totally negative by IFA (data not shown).

Electron microscopy analyses performed on cultured cells with a strong CPE demonstrated the presence of multinucleated giant cells. Typical foamy viral particles (of 100–110 nm of diameter) were frequently observed, with several envelope spikes and a spherical central core (figure 2B). Budding of such viral particles was mainly observed from membrane surface of the endoplasmic reticulum, as known for such infection [19,35,36].

### Molecular results

High molecular DNA was obtained from the peripheral blood buffy-coat of 49 out the 56 animals with a total of 95 DNA samples obtained during the follow-up. Among the 49 monkeys, there were 21 SFVs seropositive and 28 seronegative animals respectively. In 7 monkeys, (including 4 SFV seropositive), buffy-coat was not available. Nested polymerase chain reaction for the LTR and the *integrase *regions were performed on 49 DNAs corresponding to the most recent obtained sample, from the 49 animals (Table 1).

**Figure 3:**
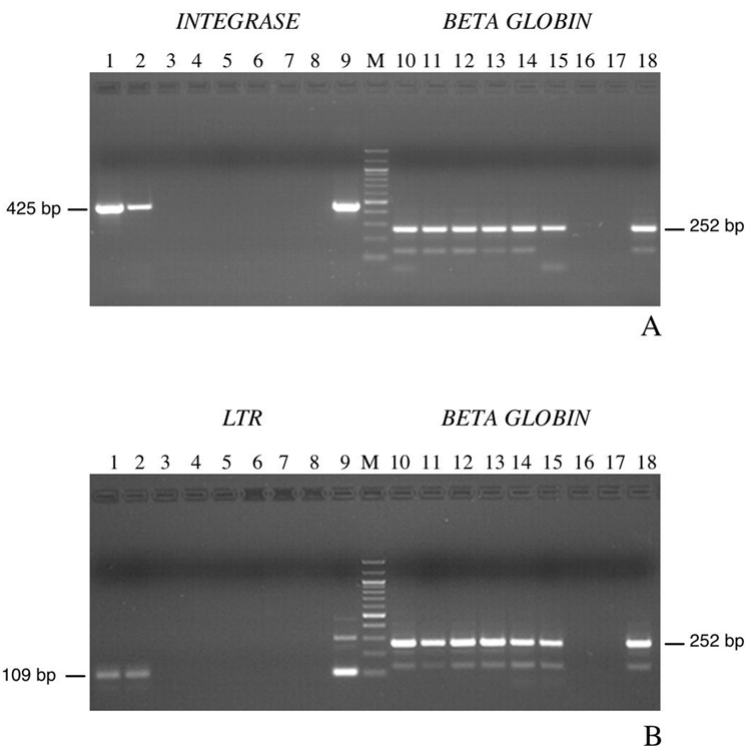
**Semiquantitative PCR for SFV **a) Study of *integrase *and the Beta globin genes in MtoT2 DNA. Lane 1–7 and 10–16: serial dilutions of the DNA from 500 ng to 0,5 pg. Lanes 8 and 17: negative controls. Lanes 9 and 18: positive controls. M: 100 bp ladder b) Study of LTR and Beta globin genes in MtoT4 DNA. Lane 1–7 and 10–16: serial dilutions of the DNA from 500 ng to 0,5 pg. Lanes 8 and 17: negative controls. Lanes 9 and 18: positive controls. M: 100 bp ladder

All the DNA samples (n = 29), originating from SFVs seronegative monkeys, scored PCR negative. By contrast, as seen in Table 1, 18 DNA samples, out the 21 SFVs seropositive animals, scored positive with the *integrase *and/or LTR PCR. Only 4 DNA samples were found positive for both nested PCR assays. To determine whether these discrepancies of results between the two PCR assays could be related to a low viral load (reaching the limits of our PCR sensibility), we used a semi-quantitative PCR. Fifteen out of the 18 positive monkeys had a very low viral load, ranging from 1 to 10 copies in 500 ng of total DNA. In only three cases (two of them being positive for both nested PCRs), the SF viral load reached 100 copies in 500 ng of total DNA (figure 3 and Table 1).

Apart from the 15 *integrase *positive samples obtained from DNAs of the buffy-coat (Table 1), we also obtained by PCR two other similar fragments from the cultured cells of two FVs seropositive animals (T6 and TG1), whose uncultured peripheral blood cells were found negative by PCR.

### Genetic variability of foamy viruses

#### Overall genetic variability

The 17 samples, found *integrase *positive, were cloned and one clone for each of them was sequenced. Genetic comparison of these 17 new SFVs strains between themselves showed that 14 belonged to 3 main molecular groups (that we called *TM*A, *TM*B, *TM*C). In addition, 3 sequences that we called *TM*D, *TM*E and *TMF*, did not belong to these 3 groups. As seen in Table 3, the strains originating from TQ3, TD3, T1, TG2 and TG3 (*TMA *group) were nearly identical to each other (99.5 to 100% at the nucleotide level) as were the three sequences from T4, T7 and TF2 (*TMB *group) that exhibited 99.5 to 100% similarity. Furthermore, the six sequences from TI4, T5, TK3, T6, TG1, and TM3 (*TMC *group) were also nearly identical (99.7 to 100%). Finally, the three last sequences originating from Z10 (*TMD*), T2 (*TME*) and TD1 (*TMF*) were different to each other, as well as to the 14 other ones (Table 3). Thus, members of this colony of *Macaca tonkeana *were infected by 6 different strains of SFVs. Divergences ranged from 5.5% to 17.4% at a nucleotide level between these genetic clusters.

**Table 3:** Percent of nucleotide identities between the 17 new *Macaca tonkeana *sequences and 6 other published prototypic FVs sequences from macaques. The comparison was based on a fragment of 425 bp of the SFV *integrase*. We showed the 6 different groups of SFV strains (A to F) characterized in this study.

	A					B			D	E	C						F						
												
	*MtoTQ3*	*MtoTD3*	*MtoT1*	*MtoTG2*	*MtoTG3*	*MtoT4*	*MtoT7*	*MtoTF2*	*MtoZ10*	*MtoT2*	*MtoTI4*	*MtoT5*	*MtoTK3*	*MtoT6*	*MtoTG1*	*MtoTM3*	*MtoTD1*	*MmuSFV Mac*	*Mmu SFV1b*	*Msi Sophie*	*Mne PT310*	*Pne 50057*	*MarHeb*
*MtoTQ3*	**100**	**100**	**100**	**99,76**	**99,76**	91,43	91,19	91,19	89,76	89,76	85,24	85,24	85,24	85,24	85,24	85	90,11	89,76	88,33	88,70	90,58	90,11	95,76
*MtoTD3*	**100**	**100**	**100**	**99,76**	**99,76**	91,43	91,19	91,19	89,76	89,76	85,24	85,24	85,24	85,24	85,24	85	90,11	89,76	88,33	88,70	90,58	90,11	95,76
*MtoT1*	**100**	**100**	**100**	**99,76**	**99,76**	91,43	91,19	91,19	89,76	89,76	85,24	85,24	85,24	85,24	85,24	85	90,11	89,76	88,33	88,70	90,58	90,11	95,76
*MtoTG2*	**99,76**	**99,76**	**99,76**	**100**	**99,52**	91,19	90,95	90,95	89,52	89,52	85	85	*85*	85	85	84,76	89,98	89,52	88,1	88,47	90,35	89,88	95,52
*MtoTG3*	**99,76**	**99,76**	**99,76**	**99,52**	**100**	91,19	90,95	90,95	89,52	89,52	85	85	85	85	85	84,76	89,98	89,52	88,1	88,47	90,35	89,88	95,52
*MtoT4*	91,43	91,43	91,43	91,19	91,19	**100**	**99,76**	**99,76**	94,52	92,14	85,24	85,24	85,24	85,24	85,24	85	91,52	91,9	91,19	90,58	95,76	91,05	91,76
*MtoT7*	91,19	91,19	91,19	90,95	90,95	**99,76**	**100**	**99,52**	94,29	91,9	85	85	85	85	85	84,76	88,47	91,67	90,95	90,35	91,76	88,94	89,41
*MtoTF2*	91,19	91,19	91,19	90,95	90,95	**99,76**	**99,52**	**100**	94.29	92,38	85	85	85	85	S5	84,76	91,29	91,67	90,95	90,35	95,52	90,82	91,52
*MtoZ10*	89,76	89,76	89,76	89,52	89,52	94,52	94,29	94,29	100	91,19	84,29	84,29	84,29	84.29	84,29	84,52	90,35	91,67	90,24	91,29	94,35	90,58	90,11
*MtoT2*	89,76	89,76	89,76	89,52	89,52	92,14	91,9	92.38	91,19	100	82,86	82,86	82,86	82,86	82,86	82,62	92,23	93,57	89,29	88,47	91,29	92,94	89,41
*MtoTI4*	85,24	85,24	85,24	85	85	85,24	85	85	84,29	82,86	**100**	**100**	**100**	**100**	**100**	**99,76**	83,76	84,05	85,71	88,47	84,70	83,76	83,52
*MtoT5*	85,24	85,24	85,24	85	85	85,24	85	85	84,29	82,86	**100**	**100**	**100**	**100**	**100**	**99,76**	83,76	84,05	85,71	88,47	84,70	83,76	83,52
*MtoTK3*	85,24	85,24	85,24	85	85	85,24	85	85	84,29	82,86	**100**	**100**	**100**	**100**	**100**	**99,76**	83,76	84,05	85,71	88,47	84,70	83,76	83,52
*MtoT6*	85,24	85,24	85,24	85	85	85,24	85	85	84,29	82,86	**100**	**100**	**100**	**100**	**100**	**99,76**	83,76	84,05	85,71	88,47	84,70	83,76	83,52
*MtoTG1*	85,24	85,24	85,24	85	85	85,24	85	85	84,29	82,86	**100**	**100**	**100**	**100**	**100**	**99,76**	83,76	84,05	85,71	88,47	84,70	83,76	83,52
*MtoTM3*	85	85	85	84,76	84,76	85	84,76	84,76	84,52	82,62	**99,76**	**99,76**	**99,76**	**99,76**	**99,76**	**100**	83,52	83,81	85,48	82,35	84,47	83,52	83,29
*MtoTD1*	90,11	90,11	90,11	89,98	89,98	91,52	88,47	91,29	90,35	92,23	83,76	83,76	83,76	83,76	83,76	83,52	100	92,7	89,6	88	90,58	91,05	89,17
*MmuSFVMac*	89,76	89,76	89,76	89,52	89,52	91,9	91,67	91.67	91,67	93,57	84,05	84,05	84,05	84,05	84,05	83,81	92,7	100	88,57	88,94	91,76	92,23	89,88
*MmuSFV1b*	88,33	88,33	88,33	88,1	88,1	91,19	90,95	90,95	90,24	89,29	85,71	85,71	85,71	85,71	85,71	85,48	89,6	88,57	100	88,47	90,82	88,94	87,76
*Msi Sophie*	88,70	88,70	88,70	88,47	88,47	90,58	90,35	90,35	91,29	88,47	88,47	88,47	88,47	88,47	88,47	82,35	88	88,94	88,47	100	91,29	88,47	88,94
*MnePT310*	90,58	90,58	90,58	90,35	90,35	95,76	91,76	95.52	94,35	91,29	84,70	84,70	84,70	84,70	84,70	84,47	90,58	91,76	90,82	91,29	100	90,82	91,52
*Pne50057*	90,11	90,11	90,11	89,88	89,88	91,05	88,94	90,82	90,58	92,94	83,76	83,76	83,76	83,76	83,76	83,52	91,05	92,23	88,94	88,47	90,82	100	89,17
*MarHeb*	95,76	95,76	95,76	95,52	95,52	91,76	89,41	91,52	90,11	89,41	83,52	83,52	83,52	83,52	83,52	83,29	89,17	89,88	87,76	88,94	91,52	89,17	100

To confirm these results, we decided to analyse also the LTR of these SFVs. However, as the length of the LTR fragment amplified in our study is too small (109 bp) for reliable phylogenetic analyses, we decided to amplify our DNA samples using the LTR primers described by Engel et al [14], which generate a 336 bp fragment. Thirteen out of the 18 PCR positive (for *integrase *and/or LTR regions) showed a positive result. We found a perfect concordance for all the strains with the same molecular groups as previously identified using the *integrase *sequences: MtoT1, MtoTG2, MtoTG3, MtoTQ3 and MtoTD3 form a group (the *TMA *group), MtoTK3, MtoTG1, MtoT6 and MtoT5 form another group (the *TMC *group) and finally MtoTF2 and MtoT7 form the *TMB *group (data not shown).

Genetic comparison of the 17 new 425 bp *integrase *sequences with all the other available SFVs *integrase *sequences indicated that they exhibited from 62,1% to 95,8% of similarity at the nucleotide level with the different other SFVs strains. As seen in Table 3, it is worthwhile to note that the only 11 available *integrase *genes from other macaque species (including the prototypes *MmuSFV1b, McySFV2, MmuSFVmac*) were neither identical, nor very closely related (4.2% to 16.7% of nucleotide divergence) to the new sequences from *M. tonkeana*, obtained in this study.

#### Intra-strain genetic variability

To look for the intra-strain genetic variability of such SFVs *in vivo*, we sequenced 10 clones of the *integrase *gene fragment obtained from a PCR performed with 2 different DNA samples (Z10 and TQ3). The results showed 3 and 5 mutations respectively for the 2 series of 10 clones, indicating a very low intra-strain genetic variability (8/8500 = 1°/°°).

#### Overtime genetic variability

To gain new insights into the overtime genetic variability of such SFVs in a same individual, we amplified by PCR 17 DNA samples originating from 7 animals followed with a mean time of 6 years and 5 months (range 2 to 12 years). One clone was sequenced for each *integrase *PCR sample. In 4 cases, the sequences of the *integrase *gene fragment of 425 bp were totally identical, while in the 3 other monkeys, only one base (in two cases) and 4 bases (in one case) were observed in samples originating from the same animal.

### Phylogenetic analyses

A comprehensive phylogenetic study was performed with the Neighbor-Joining method using the 17 novel SFVs sequences generated in this study, and all 11 other *integrase *gene fragments from Asian monkeys, available in GenBank. We also included in this analysis 31 prototypes of SFVs from Asian and African apes and from African monkeys. The strain ApsSFV8spm originating from a South American spider monkey was used as out-group to root the tree.

**Figure 4:**
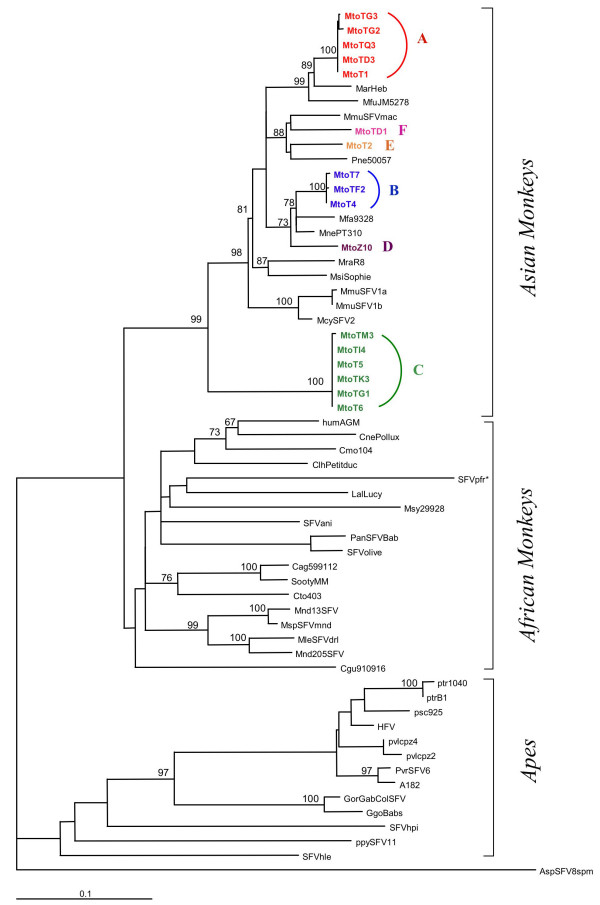
**Phylogenetic tree generated on a 425 bp fragment of the *integrase *FV gene**. The tree includes all of the 17 new *macaca tonkeana *FV described in this study and other FV sequences from African and Asian apes and monkeys available in GenBank. The phylogeny was generated with the Neighbor-joining method, performed in the PAUP program (v4.0b10). The sequence alignment was submitted to the Modeltest program (version 3.6) to select the best model to apply to phylogenetic analyses. The selected model was the GTR+G+I one. The reliability of the inferred tree was evaluated by bootstrap analysis on 1000 replicates. Numbers at each node indicate the percentage of bootstrap samples in which the cluster to the right is supported and only values greater than 60% are shown. The branch lengths are drawn to scale with the bar indicating 0.1 nucleotide replacement per site. The tree was rooted by using the New World spider monkey Asp(SFV8spm) sequence. *= SFVpfr: (*Presbytis Francoisi*): despite the Asian origin of this monkey, its sequence clusters within the large African Monkey clade.

As seen in figure 4, there are three main SFVs clusters. The first one comprising the sequences from Apes, the second one corresponding to the sequences from the African monkeys and the third one comprising all the sequences from Asian monkeys. As expected, the 17 novel sequences from *M. tonkeana*, generated in this study, were clearly located within the large and highly phylogenetically supported Asian clade (99% bootstrap value). Within this Asian group, two main groups supported by high bootstrap values could be identified. The first one (*TMC*-bootstrap of 100%) corresponds to a group of 6 new sequences from *M. tonkeana*. The second group (bootstrap of 98%) comprised all the other 22 Asian SFVs sequences. Within this second clade, several sub-clusters that are highly supported phylogenetically (bootstrap of 73–100%) could be identified and two of them comprised only *M. tonkeana *sequences (these two groups are *TMA *and *TMB*).

### Modes of transmission of SFVs in this colony

#### Very little evidence of SFVs transmission from mother to child and between siblings

Based on serological findings, there is only very little evidence for a mother to child transmission of SFVs. Indeed, in this series, all but one of the 21 immatures, were seronegative for foamy viruses at their first sample and remained negative until at least 3 years, despite the fact that their mother was infected in all but two cases, when she gave birth to each of them. This contrasts sharply with the situation for STLV-1. Indeed, in this case, most of the immatures are infected by STLV-1 (probably through breast-feeding) at their first sample and the only STLV-1 seronegative immature had an STLV-1 seronegative mother.

When considering the molecular results, 8 out 11 mother and child infected pairs are infected by different viral strains. Furthermore, none of the 7 pairs of infected siblings harbored a similar virus between themselves. Furthermore, regarding father to child transmission, it is interesting to note that among the 7 children of T1 (verified by genetic exclusion of paternity), 3 different strains of SFVs are present. All these data argue against a significant transmission of simian foamy viruses from mother or father to child as well as between siblings.

#### Evidence for acquisition of SFVs infection during severe bites, mainly in sub-adults or young adults

On a serological point of view, it is worth noting that the SFV seroconversion followed the first documented important episode of severe bite (with a dermal wound) in 7 out of 10 animals. For example, in case of T9, we observed a seroconversion between 1991 and 1993 and the first severe injury was registered in 1992. Furthermore, for TG1, the seroconversion was observed between 1996 and 2002 and the first important injury was declared in 1998.

On a molecular point of view, the situation is less clear, especially because it is very difficult in the case of a severe wound to know exactly whose animal is responsible for the bite. However, one case is particularly informative: during the year 2003, TD3 and TG2 had frequent conflicts and TD3 received severe bites. TD3 was negative in 2002 and was found to be positive in 2004. Interestingly, TD3 was infected by the *TMA *strain identical to that found in TG2 in 2002.

## Discussion

Our findings on the FV prevalence confirmed that captive colonies of non-human primates are often highly endemic for foamy viruses [4,16,20]. In fact, in our study, nearly half of the animals (and 89% of the adults) are infected by FVs. Furthermore, for the first time, we extended herein to *M. tonkeana *species the presence of such high levels of FVs infection.

It is important to note that our study took place in a quite large, free-breeding colony, which is not the case in most of the few other studies performed for FVs in NHP captive colonies [2,4,8,20,22]. Moreover, some of the published studies have been performed in colonies comprising different species of monkeys. For instance, in a captive US colony of 254 baboons, including 150 adults and 104 juvenile, 88% (132/150) of the adults were FVs seropositive. However, this colony comprised at least 3 different subspecies of baboon (*Papio ursinus, P. anubis *and *P. cynocephalus*) of different origins, with some animals having been recently captured in East Africa, while most of the other baboons were long time residents of the captive colony (5 to >15 years). Furthermore, in this group, most of the young animals were removed from the breeding harem at 6–9 months of age, therefore reducing the opportunities of being infected by FVs [2,28]. In another US colony of baboons, all the 38 adults, housed in gang cages, were found FVs seropositive while all but one of the 10 juveniles, were found seronegative. However, in this work too, the juveniles were not housed in most of the cases with their mothers, having been removed from them shortly after birth [20].

Regarding specifically Asian monkeys, a survey of a colony of *M. fascicularis*, held at Health Canada (Ottawa), and all bred from wild-caught animals, indicated that 80% of the 395 animals were infected by SFVs [8]. Verschoor et al., found also that 69.4% of 108 orangutan blood samples originating from a reintroduction center in East Kalimatan were found seropositive for FVs [37]. Lastly, only one study recently published has been performed in a free-ranging colony of monkeys, i. e. a group of 38 macaques (mostly adults) living in a temple in Central Bali, Indonesia. In this case, the seroprevalence for FVs was of 89.5%, reaching 93% in adults [14].

In our study, we have also analyzed the presence of FVs proviral DNA in the peripheral blood buffy-coat DNA of most of the studied animals. The PCR negativity obtained in some animals with a clear positive WB, was probably linked to a very low viral load in the peripheral blood buffy-coat (< than 1–10 copies in 500 ng DNA, i.e. 75 000 cells). Moreover, we can strongly suggest here that the negativity of PCR is not due to the presence of divergent foamy viruses, but to a low viral load. Indeed, in two animals, T6 and TG1, for which the *integrase *PCR was negative in the uncultured PBMCs, we were able to amplify the same *integrase *fragment on cultured cells. Up to know, little is known about the FVs proviral load in naturally infected NHPs [28]. In a study of African green monkeys, a very low proviral load was detected in most tissues and very recently, a report described that SFV DNA was present at a low copy number in PBMCs and tissue from macaques [27,28]. Previously, we found, using a similar semi-quantitative technique, a low proviral load ranging from 1 to 100 copies for 500 ng of peripheral blood buffy-coat DNA, in a series of wild-caught chimpanzees [38]. Furthermore, such lack of detection of FVs sequences has also been reported in the PBMCs DNA of several hunters, living in remote villages of South Cameroon, who where found to exhibit a clear FV WB seroreactivity with the presence of the gag doublet [13].

Analysis of the foamy viral sequences found in the 17 monkeys, for which the *integrase *gene could be amplified, indicates clearly the presence of 6 different groups of FVs strains in this colony of *M. Tonkeana*. This colony has been carefully followed for more than 24 years for behavioral investigations. Furthermore, these animals have never been in contact with any other monkeys since their arrival in France in 1972 and they have neither been used in any biomedical experiments, nor injected with any biological materials [39]. Keepers did not manipulate animals from different species of the center with the same gloves. Cross-species transmission in the primatology center was thus very unlikely, but of course, it cannot be ruled out with 100% certainty. Thus, the viruses currently present in this colony should have been present originally in some of the founders of the colony. As all the members of this troop originated from only 5 different animals, this means that some of these monkeys (or at least one) should have been, at a given time, infected by more than one foamy viral strain. These different viruses were subsequently disseminated by natural means in other animals of the colony.

Concerning the possibility that the Tonkean macaques may have become infected, prior to arrival at the primate center, with SFVs from other primates species which they are sympatric with, within Sulawesi, it is important to note that the seven different taxa (species or subspecies, according to the different current classifications) of macaques present in Sulawesi are allopatric, which exclude any transmission between two different species in a given region [40]. However, the exact geographical origin of some of the founders is not known with great precision within Sulawesi Island (central or peripheral region of the distribution area of the *M. Tonkeana*). Thus, we cannot totally exclude a contamination in the wild (prior to the arrival in the primatology center) of some of the founders with a foamy viral strain originating from a related species or subspecies.

By using classical nested PCR methods for the *integrase *gene, as previously described, we did not find in any of the studied animals, a clear evidence for multiple infections by different foamy viruses. This is based on the following arguments: 1) We always found the same strain in the molecular follow-up of a specific given animal overtime and this even after 12 years of *in vivo *evolution. 2) In two animals, we sequenced ten clones of a PCR experiment and only one strain was amplified for a given animal. 3) The viruses isolated after cultures of PBMCs were identical to that found in the uncultured PBMCs of the monkeys. However, search for multiple infections in these animals are ongoing, using very sensitive PCR methods as previously described [41].

The presence of different strains of FVs in a colony, as found in the *M. tonkeana *troop, is not without precedent. Indeed, Schweizer et al. found in a troop of 19 African green monkeys (originating from Kenya), and living together in a monkey house, four different FVs clusters with high homologies (>95%) in the envelope surface domain gene [22]. Between the clusters, the divergences ranged from 3 to 25%, indicating thus that four different strains or subtypes of simian FVs were prevalent in this colony. In another study, Blewett et al., have shown the presence of 2 different FVs (based on *pol *and LTR sequences) in a colony of baboons [2]. However, these two distinct clades consisted of isolates from yellow and olive baboon and isolates from chacma baboons respectively. Very recently, Jones-Engel et al., found in *M. tonkeana *the presence of at least 4 different strains of FVs [14]. This observation was based on the analysis of a small fragment of the LTR and some of the observed clades were not clearly supported phylogenetically (low bootstrap values).

The exact modes and timing of SFV transmission in monkeys is unknown although both sexual and oral transmission have been suspected [20,27]. Furthermore, it is rather difficult to compare our results with those of the very few other published studies with a follow-up as they were performed, as seen above, in different kinds of colony with sometimes the immatures being removed from their mother, either shortly after birth or after few months [2]. Moreover, modes and timing of infection for a specific virus can vary according to the different behaviors of different monkey or apes species studied, as well demonstrated in the case of STLV-1 [42-44]. Here, the possibility to study a free-breeding colony of monkeys with long-term follow-up with both plasma and DNA sequential samples and behavioral investigations provide us a unique work opportunity.

We provided here serological and molecular data arguing against a significant transmission of simian foamy viruses from mother or father to child as well as between siblings.

Concerning a possible sexual acquisition of SFV, it is interesting to note that the seroconversion timing of SFVs strongly contrasted with that found for the herpes B virus, whose primary mode of infection is sexual contact [45]. In this colony of *M. tonkeana*, most males become positive for herpes B between 2.5 and 6 years of age because they may early start to mount adult females (outside their fertility period), whereas females seroconverted only after puberty, i.e. from 5 years of age (unpublished data from Strasbourg Primatology Center). With regards to foamy viruses, a majority of individuals remained negative until 7 years of age making thus improbable, mounts as a possible way of transmission.

Most cases of seroconversion for foamy viruses occurred when individuals reached adulthood, a period of life that entails an increased likelihood of biting. After 7 years of age, for instance, males entered in competition for access to oestrous females and they occasionally received wounds from their rivals [40]. Indeed, it is clear that in this colony, SFVs seroconversion followed the first important recorded episode of severe bite (with a dermal wound) in 7 out of 10 animals. Concerning the molecular point of view, it is very difficult in case of a severe wound to know exactly who is the animal responsible for the bite. However, in our colony, we can demonstrate the direct transmission of a specific FV strain from a positive to a seronegative animal after an episode of severe bites. All together, these data suggest strongly natural transmission of SFVs via severe bites with contact of saliva from the infected animal to the blood strain of the recipient. However, as viral loads have been shown to be very important determinants for transmission of other primate retroviruses, more conclusive evidence for SFVs transmission routes in primates will require determination of viral loads in different body fluids such as saliva, semen, vaginal lavages and breast milk.

Our findings fit very well with studies demonstrating that SFVs are present in the saliva of infected macaques and baboons [2,28] and that oral tissues are important site for FV replication in African green monkeys and macaques [27]. Furthermore, it is interesting to note that most of the SFV infections, reported in persons occupationally exposed to non-human primates in zoos or primates centers, have probably been acquired through bites [8-12,14,46]. Very recently, natural acquired SFV infections have also been found in few hunters in Cameroon, central Africa [13] and in an ongoing study, we could demonstrated that bites from a monkey or an ape is, in central Africa, a major risk factor for acquiring such SFV infection ([47] and Calattini et al., in preparation).

## Methods

### Animals

A *Macaca tonkeana *captive colony, housed in the Strasbourg Primatology Center, was investigated for the presence of simian foamy viral infection. This colony was established originally from 7 animals, all originating from the central part of Sulawesi (Indonesia) and brought to the Strasbourg center in 1972. From 1972 to 1978, these animals were housed together. Only one (MtoT2) of the seven founders was still alive when we began this work more than twelve years ago. In 1978, the colony was separated in two groups, the first comprising only 3 animals, which constituted the original nucleus (or founders) of the current studied colony. Lastly, two animals from the second group were incorporated in the colony; one in 1981 (MtoT10) and one in 2002 (MtoZ10). Since then, the progeny has been maintained in large wooded enclosure at the primatology center. The animals have been carefully followed for behavioral investigations for 24 years. During this long period of follow-up, animals were visited every day by an ethologist who controls the animal status and especially the presence or absence of wounds or bleedings. Furthermore, the animals were never in contact with other monkey species and were never used in biomedical experiments, nor infected with any biological material [34,39].

### Serological tests

All plasma samples were first analyzed to investigate the presence of FVs antibodies as previously described [4,5,48]. Briefly, a Western Blot (WB) assay was performed using, as a source of foamy viral antigens, a BHK-21 cell line infected with a chimpanzee SFV strain. Plasma were tested at 1:100 dilution. WB seropositivity was defined as the presence of a clear reactivity to the Gag doublet of 70 and 74 KDa. To validate our results obtained with a chimpanzee antigen, we also tested a large subset of samples with a WB using, as viral antigen, a lysate of BHK-21 cells infected by the MtoT6 virus, which originated from a monkey of the *M. Tonkeana *colony. The WB conditions were the same as previously described.

### Virus isolation

Virus isolation was done in animals showing a strong WB seropositivity, as previously described [5,7,11,35]. Briefly, BHK-21 cells were maintained in DMEM medium supplemented with 5% of fetal calf serum (FCS) and antibiotics. Fresh blood samples were collected in EDTA tubes and PBMCs (Peripheral Blood Mononuclear Cells) were isolated on Ficoll-Hypaque gradient. PBMCs were then maintained for 2 days in RPMI medium containing 20% FCS, antibiotics and phytohemagglutinin (PHA) at 3 μg/ml and further stimulated with IL-2 (100U/ml). After 4 days of stimulation, PBMCs were co-cultivated with BHK-21 cells. Cultures were checked daily for syncytial cytopathic effect (CPE) typical of FV infection.

For transmission electron microscopy, cells were fixed in 2.5% glutaraldehyde and 1% paraformaldehyde in 0.15 M cacodylate buffer complemented with MgCl_2_, CaCl_2 _and sucrose at 0.1 M. After 2 days at 4°C, the filters were washed during 2 hours in cacodylate buffer and treated with 1% of osmium teroxide solution and 1% potassium ferrocyanide for 1 hour at room temperature. Cells were dehydrated in ethanol and included in an epoxy resin at 60°C for 48 hrs. Ultrathin sections were performed on a microtome Leica ultracut UCT. Sections were then examined in a Jeol 1200 EX electron microscope.

### Indirect immunofluorescence

An indirect IF assay was performed on co-cultivated cells at 7 and 21 days post- infection. The primary antibody of the reaction was a serum derived from a rabbit experimentally infected with a chimpanzee SFV strain; the secondary antibody was a fluorescein-conjugated goat anti-rabbit diluted 1:500. Cells were then mounted with DAPI-containing mounting medium and visualized with a Zeiss Axioplan 2 imaging microscope X40 using a Zeiss Axiocam Hrc (color) camera and the Zeiss Apotome software. For each reaction, a negative and positive control was added. The positive control corresponded to BHK-21 cells infected with a chimpanzee SFV strain [36], while the negative control consisted of uninfected BHK-21 cells.

### Molecular studies

High molecular weight genomic DNA was extracted from the buffy-coat of the studied animals and of several positive and negative controls using the Qiagen kit (QIAmp blood Mini Kit, Courtaboeuf, France). Two SFV proviral genomic regions (a 425 bp fragment of the *integrase *gene and a 109 bp fragment of the LTR) were studied using generic, nested primers as previously reported [5,21]. The presence and quality of the extracted DNA were verified by amplifying a ß-globin gene fragment.

In order to calculate the sensitivity of the two nested assays, DNA was extracted from a cell line (HFV-2) containing 2 copies of integrated foamy virus genome and then amplified with a semi-quantitative PCR. The sensitivity of our tests ranged from one to 10 copies detected in 500 ng (75 000 cells) of cellular DNA. We estimated the viral load in samples that showed a positive result after the qualitative PCR assay. Thus, we performed a semi-quantitative PCR by amplifying six 10-fold serial dilutions of the DNA ranging from 500 ng to 0,5 pg. The PCR conditions and the cycling were performed as previously described [4,21]. Each sample was amplified separately for the ß-globin gene and for the viral target. The quantification of the viral load was expressed as the number of viral genome in 500 ng of total DNA (i.e. 75000 cells). *Integrase *PCR products were purified, cloned in a pCR vector and sequenced using the BigDye terminator cycle kit and an ABI 3100 automated sequencer (Applied Biosystem). The 17 new *integrase *gene fragments sequences of simian foamy viruses determined herein were deposited in the National Center for Biotechnology Information database. The GenBank accession numbers are DQ354073 to DQ354089.

### Phylogenetic analyses

Multiple nucleotide sequences alignment was performed with the DAMBE program on the basis of a previous amino-acid alignment created from the original sequences. The final alignment was submitted to the Model Test program to select the best phylogenetical model to apply for the phylogentical analyses. The best phylogenetical model, selected using Model Test was the GTR+I+G model (-lnL= 6502.5806) with a shape of 0.9959 and a pinvar of 0.2901. The phylogeny was derived by the Neighbour-Joining method (with a bootstrap value of 1000), performed in Paup program [49,50].

### HTLV-1/STLV-1 serology

All sera or plasma were tested for STLV-1 antibodies by an immunofluorescence assay (IFA) with HTLV-1 (MT2) or HTLV-2 (C19) producing cell lines, as previously described [32]. Furthermore, all samples were tested by a Western Blot, which contains disrupted HTLV-1, a recombinant protein (RGD21) that reacts with both HTLV-1 and HTLV-2 antibodies and the two gp46 peptides MTA1 and K55 [32].

## Abbreviations

SFV: Simian Foamy Virus

HIV/SIV: Human Immunodeficiency Virus/Simian Immunodeficiency Virus

NHP: Non-Human Primates

STLV/HTLV: Simian T Lymphotropic Virus/Human T Lymphotropic Virus

WB: Western Blot

PBMC: Peripheral Blood Mononuclear Cell

CPE: Cytopathic Effect

IFA: Immunofluorescence Assay

PCR: Polymerase Chain Reaction

## Competing interests

The author(s) declare that they have no competing interests.

## Authors' contributions

SC performed the laboratory work. FW, BT and NH provided all of the samples, information on the colony as well as the long-term follow up of behavioral observations and revised critically the manuscript. CS carried out the electron microscopy. SB did the STLV serological assays. AS helped to western blot assays and revised critically the manuscript. AG coordinated the study, participated to the obtention of the samples and wrote the manuscript. All authors read and approved the manuscript.
